# Mindful Kangaroo Care: mindfulness intervention for mothers during skin-to-skin care: a randomized control pilot study

**DOI:** 10.1186/s12884-021-04336-w

**Published:** 2022-01-15

**Authors:** Marc-Antoine Landry, Kumar Kumaran, Juzer M. Tyebkhan, Valerie Levesque, Marcello Spinella

**Affiliations:** 1 Department of Pediatrics, University of Alberta, Edmonton, Canada; 2 Stollery Children’s Hospital, Newborn Intensive Care Unit, Royal Alexandra Site, Edmonton, Canada; 3 Edmonton NIDCAP Training Centre Canada (ENTCC), Edmonton, Canada; 4 School of Social and Behavioral Sciences, Stockton University, Galloway, NJ USA

**Keywords:** Mindfulness, Skin-to-skin care, Kangaroo Care, Maternal stress, Depression, Anxiety

## Abstract

**Background:**

Parents of babies admitted to the Newborn Intensive Care Unit (NICU) undergo considerable stress. There is evidence that mindfulness reduces stress in these parents. Kangaroo Care (KC) is practiced in NICUs across the world and is stress-relieving. Whether mindfulness practiced during KC in the NICU reduces parental distress has not yet been studied. The objective was to explore the feasibility and acceptability of teaching and practicing mindfulness during KC for mothers of premature babies. The objective was also to document preliminary outcomes of Mindful Kangaroo Care (MKC) on maternal stress, anxiety, depression, and mindful awareness.

**Methods:**

In this pilot randomized controlled study, mothers of premature babies who were expected to stay in the NICU for at least four weeks were taught two mindfulness exercises to practice during KC and compared to mothers who received standard care with no mindfulness teaching. Mothers filled out stress, anxiety, depression and mindful awareness scales at recruitment and after four weeks. Acceptability and feasibility questionnaires were also completed.

**Results:**

Fifteen mothers per group completed the study. The MKC group demonstrated a significant within-group reduction in anxiety (*p* = 0.003), depression (*p* = 0.02) and stress (*p* = 0.002), and a significant increase in both the curiosity (*p* = 0.008) and decentering (*p* = 0.01) scores of the Toronto Mindfulness Scale, all of which had medium to large effect sizes. Only the increases in curiosity and decentering were significant between groups. Fourteen mothers found the intervention acceptable, one neutral.

**Conclusion:**

MKC was acceptable, feasible and led to a reduction in stress, anxiety and depression in mothers who practiced mindfulness exercises during KC.

**Supplementary Information:**

The online version contains supplementary material available at 10.1186/s12884-021-04336-w.

## Background

Parents of babies admitted to the Newborn Intensive Care Unit (NICU) undergo considerable stress [1]. Symptoms of acute stress and post-traumatic stress disorder have been reported between 28 and 51% of parents [2, 3]. NICU parents also report a high level of mood and anxiety symptoms across diverse ethnocultural groups and countries [4].

Mindfulness offers a way to focus on being in the moment, by accepting the present moment in a non-judgmental manner [5]. Mindfulness has been shown to be feasible in the NICU [6–9] and reduced parental stress [7, 8]. Parents generally want to remain with their babies when in the NICU and do not attend group programs [8]. When mothers provide Kangaroo Care (KC), they are with their babies and generally available to receive information. Bringing mindfulness to the bedside and teaching this to mothers during Kangaroo Care has not, to our knowledge, been investigated.

Kangaroo Mother Care was developed to complement neonatal care for premature and low birth weight infants in resource-poor settings [10, 11]. During KC, a baby is positioned in skin‐to‐skin contact with the mother's (or father's) chest for varying periods of time [12]. KC is widely practiced across the world and has been shown to reduce maternal stress, anxiety and depression [12, 13]. KC for the very preterm or very low birth weight can be challenging for babies, parents and staff [14, 15].

We hypothesized that mindfulness during KC would be feasible and acceptable to the mothers. We also hypothesized that practicing mindfulness during KC would reduce maternal stress, anxiety and depression, and would increase mindful awareness.

## Methods

This study was a non-blinded, prospective, observational, pilot randomized controlled study. Mothers with preterm babies admitted to the Stollery Children Hospital NICU, Royal Alexandra site, were eligible to enter the study if (a) fluent in spoken and written English, (b) the baby had an anticipated stay at this NICU for at least four weeks following recruitment, (c) if the baby's medical condition allowed KC at recruitment, (d) if the mother was available for regular KC during the four weeks after recruitment, and (e) if mother agreed to receive teaching for 30–45 min weekly during those four weeks. Mothers who reported current active mental illness and current or prior substance abuse/addiction were excluded. Given the pilot nature of this study, a power analysis was not done. We aimed to recruit a convenience sample of 30 participants (15 per group) [16]. The study was approved by the Regional Ethical Review Board at the University of Alberta (study code: Pro00089053). This study was retrospectively registered on January 6^th^, 2021, at clinicaltrials.gov (registration number: NCT04696770).

To optimize enrolment, the NICU Social Workers, who meet with all families in the NICU during the first days of admission, were asked to introduce this pilot study to mothers, then inform the research team of their potential interest. Once recruited, mothers were randomized into the Mindful Kangaroo Care (MKC) group or the Standard Care (SC) group. To avoid selection bias, we used block randomization using random block sizes [17]. Sealed envelopes were prepared and shuffled by one team member. Each envelope contained the group allocation for one study subject and the allocation was revealed after consent and recruitment.

### Mindfulness intervention

Mothers in the MKC group had face-to-face direct mindfulness coaching during weeks 1 and 2, and ongoing support during weeks 3 and 4. The principal investigator is a Unified Mindfulness certified lead coach [18]. During the first encounter, mothers learnt an introductory mindfulness technique to develop the awareness of any sensations felt in the body while holding their baby in KC, with an emphasis on the physical sensations created by the presence of their baby. At the second encounter, mothers learnt a mindfulness technique to create positive emotional sensations in the body triggered by holding their baby skin-to-skin. In both cases, the baby was at the centre of the mindfulness practice. Additionally, mothers had access to downloadable audio files of both techniques' instructions and guided practice sessions (duration 16 & 21 min). See Additional files 1, 2, 3 and 4 for the written script of those audio files.

### Control

Mothers in the control group received Standard Care which, in this NICU, includes KC according to bedside staff's judgement of baby and mother readiness for KC. SC mothers did not receive any mindfulness coaching but had a weekly visit to encourage them to fill out the KC log.

### Questionnaires

All mothers were asked to complete a general demographic form at enrolment and a KC Log, on which they were asked to record the timing and duration of each KC session and document concurrent activities during KC. The demographic form and the KC logs for both the intervention and the control groups developed specifically for this study are provided as Additional files 5, 6 and 7 respectively.

Mothers were asked to complete the following questionnaires at enrolment (Pre) and four weeks after enrolment (Post):

(A) To evaluate stress, mothers filled out the Parental Stressor Scale for NICU parents, a validated situation-specific scale for parents who have a baby hospitalized in NICU. Parents rate sources of stress within three domains: sights and sounds, infant behaviour and appearance, and parental role alterations—scores for each question range from 1 (not at all stressful) to 5 (extremely stressful). A higher score indicates a higher level of stress [19]. The Parental Stressor Scale for NICU parents is copyrighted and permission was obtained to use it for this study.

(B) To evaluate symptoms of depression and anxiety, mothers filled out the Patient Health Questionnaire 4, a four-item validated and standardized questionnaire to measure depression and anxiety in the general population—scores for each question range from 1 (not at all) to 4 (nearly every day). A higher score in each category suggests anxiety or depression accordingly [20, 21]. The Patient Health Questionnaire 4 is public domain and free to use for all clinical and research purposes.

(C) To evaluate mindfulness, mothers filled out the 13-item Toronto Mindfulness Scale developed and validated to measure the state of mindfulness. It measures two components: curiosity and decentering the awareness of one's experience. The curiosity questions correspond best to the awareness of the present moment, while the decentering questions correspond best to a non-judgmental attitude. The awareness of the present moment and a non-judgmental attitude are two key components of mindfulness—scores for each question range from 0 (not at all) to 4 (very much). A higher score indicates a higher level of mindful awareness [22]. The Toronto Mindfulness Scale is public domain and available to use for all research purposes.

(D) Mothers were asked to complete an acceptability form, a 3-point Likert scale created by the research team, measuring their acceptability of the different instruments and mindfulness intervention. The acceptability form created for this study is provided as Additional file 8.

The research team also maintained a feasibility form for every mother recruited with the following components: recruitment capability, data collection procedures and outcome measures, resources and ability to implement the study [23, 24]. The feasibility form developed for this study is provided as Additional file 9.

### Statistical Analysis

Study data were managed using Research Electronic Data Capture, a secure, web-based software platform hosted at the University of Alberta [25, 26]. Group demographics were compared with t-test, chi-square and Mann–Whitney U as appropriate. The scores of the three questionnaires were analyzed with the Wilcoxon Signed-Rank test for within-group comparison and the Mann–Whitney U test for between-group comparisons, given the small n and ordinal nature of the questionnaires' scores. We used the Spearman correlation test in the MKC group for correlation between the mindfulness scores and the stress, anxiety and depression scores. 2-tailed p-values were used.

## Results

Forty mothers were approached for participation from April 2019 to January 2020. Eight declined, and two withdrew after recruitment; thus, 30 mothers, 15 per group, completed the study. There were no statistically significant differences between baseline demographic data of the groups (Table 1).

**Table 1:** Mothers’ demographics

**Characteristics**	Mindful Kangaroo Care group (***n* = 15)**	Standard of Care **group (*n* = 15)**
Maternal age^a^	34 (25–42)	32 (21–41)
Parity^a^	1.4 (1–5)	2.1 (1–5)
Caesarian delivery	8	11
Gestational age (weeks)^a^	26.6 (23.1–29.4)	27.5 (24.7–30.7)
Baby's birth weight (grams)^a^	912 (555–1390)	1053 (770–1790)
Education completed (median)	College or Undergraduate	College or Undergraduate
Prior depression	7	3
Prior anxiety	6	4
Prior mindfulness	7	6
Years of prior mindfulness^a^	5.7 (1–20)	8.3 (1–20)
Prior skin-to-skin experience	1	1
Baby's age at first KC (days)^a^	3.5 (1–11)	2.6 (1–9)
Baby's age at the start of the study (days)^a^	17 (4–67)	14 (2–39)

^a^These results are expressed as mean (range)

No statistical difference between groups for any variable

All 15 MKC mothers received the two coaching sessions, and 14 had two follow-up meetings. One mother had only one follow-up meeting. Twelve SC mothers had four weekly visits, and three had three visits to encourage them to fill out the KC log.

### Kangaroo Care

Mothers recorded 337 KC sessions in the MKC group and 327 in the SC group. There were no statistical differences in time spent on concurrent activities during KC (see Table 2).

**Table 2:** Mother's activity during Kangaroo Care (KC)

	Mindful Kangaroo Care **(337 log entries)**	Standard of Care **(327 log entries)**	***p*-value**
Log entries per participant	22.5 ± 7	21.8 ± 10.4	0.84
Number of mothers who logged 4 weeks of KC	14	12	
Number of mothers who logged 1–3 weeks of KC	1	3	
KC duration (minutes)	118 ± 37	110 ± 28	0.58
Use of smartphone (% of KC sessions)	38 ± 33	30 ± 26	0.51
Sleeping (% of KC sessions)	34 ± 31	26 ± 30	0.53
Conversation (% of KC sessions)	47 ± 27	48 ± 35	0.96
Mindfulness exercise (% of KC sessions)	85 ± 21	n/a	

Results are expressed as mean ± standard deviation

Some mothers reported other activities such as reading, singing or talking. Some reported simply holding their baby

### Stress (Table 3)

There was no statistical difference between groups for the initial scores of the PSS (total and subscale scores). The MKC group showed a significant reduction in stress, from a median total score of 3.4 to 2.6 (*p* = 0.002), with 13 (87%) out of 15 mothers dropping their scores. There were no statistical differences in the total, or subscale PSS scores from enrolment to week 4, for the SC group.

### Anxiety and depression (Table 3)

The MKC group demonstrated a significant decrease in both anxiety (*p* = 0.003) and depression (*p* = 0.02) scores from recruitment to week 4. In the MKC group, 12 (80%) and 8 (53%) out of 15 mothers dropped their anxiety and depression scores, respectively. The SC group did not show any statistical differences for the change in either score, from recruitment to week 4.

### Mindfulness (Table 3)

The MKC mothers showed a significant increase in both the curiosity (*p* = 0.008) and decentering (*p* = 0.01) scores of the Toronto Mindfulness Scale, with 13 (87%) and 11 (73%) of 15 mothers reporting an increase in their curiosity and decentering scores respectively. The SC mothers did not demonstrate any changes in either score.

Within the MKC group, the degree of change in depression correlated inversely with the degree of change in mindful decentering (*r*_*s*_ = -0.68; *p* = 0.006). Correlations were also in the predicted direction for change in depression and mindful curiosity (*r*_*s*_ = -0.47; *p* = 0.08) and change in anxiety and mindful curiosity (*r*_*s*_ = -0.46; *p* = 0.08), but they did not reach statistical significance. There was no correlation between anxiety and mindful decentering (*r*_*s*_ = -0.17; *p* = 0.53).

**Table 3:** Results of questionnaires at recruitment (pre) and after four weeks (post)

	Mindful Kangaroo Care group (*n* = 15)	Standard of Care group (*n* = 15)	Between-group *p*-value
**Parental Stressor Scale**: range from 1 (not at all stressful) to 5 (extremely stressful)			
Overall pre	3.4 (2.2–4.8)	3.1 (1,7–4.4)	0.33
Overall post	2.6 (1.9–4.5)	2.4 (1.4–4.7)	
Change in Overall: Post–Pre	-0.3 (-1.7, + 0.2)	-0.1 (-1.8, + 0.7)	0.18
**Within group *p*-value** (Post–Pre)	**0.002**^**a**^	0.12	
Sights and Sounds-pre	2.2 (1.2–4.8)	2.4 (1.2–3.6)	1.00
Sights and Sounds-post	2.2 (5–17)	2.0 (1.0–4.2)	
Change in Sights and Sounds: Post–Pre	-0.4 (-1.4, + 1)	-0.2 (-1.8, + 1.2)	0.68
**Within group *p*-value** (Post–Pre)	0.051	0.21	
Infant Behavior- pre	3.5 (1.8–4.9)	2.9 (1.3–4.9)	0.22
Infant Behavior- post	2.5 (1.7–4.7)	2.2 (1.3–4.7)	
Change in Infant Behavior: Post–Pre	-0.4 (-1.9, + 0.4)	-0.2 (-1.2, + 0.6)	0.11
**Within group *p*-value** (Post–Pre)	**0.003**^**a**^	0.06	
Parental Role Alteration- pre	3.6 (1.7–5.0)	3.3 (1.5–5.0)	0.63
Parental Role Alteration- post	3.1 (1.8–5.0)	3.3 (1.3–5.0)	
Change in Parental Role: Post–Pre	-0.2 (-2.3, + 0.8)	0 (-1.8, + 2.2)	0.83
**Within group *p*-value** (Post–Pre)	0.16	0.55	
**Patient Health Questionnaire – 4**: range from 1 (not at all) to 4 (nearly every day)			
Anxiety pre	3 (1.5–4)	2.5 (1.5–4)	0.41
Anxiety post	2 (1–4)	2 (1–4)	
Change in anxiety: Post–Pre	-1 (-2.5, + 0.5)	-0.5 (-1.5, + 1)	0.09
**Within group *p*-value** (Post–Pre)	**0.003**^**a**^	0.08	
Depression pre	2 (1–4)	1.5 (1–2.5)	0.09
Depression post	1.5 (1–4)	1.5 (1–3)	
Change in depression: Post–Pre	-0.5 (-1, + 0.5)	0 (-1, + 1)	0.12
**Within group *p*-value** (Post–Pre)	**0.02**^**b**^	0.81	
**Toronto Mindfulness Scale**: range from 0 (not at all) to 4 (very much)			
Curiosity pre	1.1 (0–4)	1.8 (0–3)	0.37
Curiosity post	2.1 (0–4)	1.5 (0–3)	
Change in Curiosity: Post–Pre	+ 0.8 (-1.5, + 3.2)	-0.2 (-1.7, + 1.5)	**0.004**^**c**^
**Within group *p*-value** (Post–Pre)	**0.008**^**b**^	0.38	
Decentering pre	1.4 (0–3)	1.7 (1–3)	0.28
Decentering post	2.4 (0–4)	1.5 (0–3)	
Change in Decentering: Post–Pre	+ 0.6 (-1, + 1.4)	-0.3 (-1.7, + 0.8)	**0.005**^**c**^
**Within group *p*—value** (Post–Pre)	**0.01**^**b**^	0.23	

Results are expressed as median (range)

^a^Wilcoxon Effect size (r) is Large

^b^Wilcoxon Effect size (r) is Medium

^c^Mann-Whitney Effect size (r) is Large

### Mindful Kangaroo Care

Mothers in the MKC group reported positive experiences from the intervention. One mother, who had no prior mindfulness practice, stated: "Before this study, I felt a lot of guilt. I was constantly trying to analyze every emotion, which added a lot of stress. This study has helped me accept each emotion as it is without trying to over-analyze. My stress levels have gone down." Others commented: "I was able to block out the alarms and focus on the baby"; "The mindfulness practice impacted my experience by allowing me to let go of my stresses and connect more with my baby"; "Overall, I think I strengthened my bond with my baby and made me feel more like a mom."

### Acceptability

Fourteen out of 15 MKC mothers found the mindfulness intervention acceptable. In both groups, no mothers reported any instrument to be not acceptable (see Table 4). Two mothers were recruited in the intervention arm and withdrew from the study before completion. One mother felt overwhelmed as her twin babies were very sick, and she preferred to be alone during KC. The other mother withdrew from the study because her baby was transferred to another hospital for surgery twelve days after enrolment.

**Table 4:** Acceptability

	Mindful Kangaroo Care group (*n* = 15)	Standard of Care group (*n* = 15)
**Acceptability of questionnaires, visits, kangaroo care log**		
Acceptable	94%	89%
Neutral	6%	11%
Not Acceptable	0%	0%
**Acceptability of mindfulness interventions**		
Acceptable	93%	n/a
Neutral	7%	n/a
Not Acceptable	0%	n/a

### Feasibility

In the MKC group, the mean duration of the first and second face-to-face mindfulness coaching sessions was 30 min (range 20 to 45), and  the mean duration of the third- and fourth-week visits was 7 min (range 5 to 15 min). In the MKC group, encounters with the Principal Investigator had to be rescheduled in 32% of planned coaching or follow-up sessions for reasons related to the babies' clinical condition, the mother's health or the principal investigator's availability.

In the SC group, the mean duration of the first visit was 10 min (range 5 to 20), and almost all following three visits lasted up to 5 min.

All 30 mothers filled out the questionnaires at the beginning and completion of the study. Three babies (one in the MKC group and two in the SC group) were transferred to another hospital in the city for ongoing care after the second week because of bed capacity issues at the study site. The MKC mother had received her two coaching sessions before the transfer. All three mothers continued to keep logs of their kangaroo care sessions after transfer and had either a phone or in-person visit at the other site.

Ten (67%) out of 15 mothers listened to one or both audio files of the mindfulness technique instructions at least once during the four weeks. Eleven (73%) out of 15 mothers listened to one or both guided practices audio files. Five of the MKC and two of the SC mothers reported practicing mindfulness sporadically outside the study.

## Discussion

This study demonstrated reductions in stress, anxiety and depression symptoms in mothers of premature babies who practiced MKC for four weeks. The study also showed a significant increase in mindful awareness scores over the same period when compared to a control group.

This is the first study to explore the use of mindfulness during kangaroo care. The main strengths of the study are [a] the demonstration that mindfulness practices could be taught to mothers during a highly stressful time (i.e. while their infants are admitted to the NICU), and [b] when mothers practiced mindfulness, their stress, anxiety and depression were reduced. The study's main limitation is the pilot nature of this study, with a small sample size, which may explain why between-group statistically significant differences were not shown.

Stress significantly decreased over time for the MKC group, but the reduction did not reach statistical significance compared to the SC group. This finding was similar to the results of the pilot RCT by Petteys & Adoumie, in which only the experimental group exhibited a within-group significant decrease in the overall stress scores from enrollment to discharge, but no between-group differences were shown [7]. Mendelson et al., in a prospective observational study using mindfulness intervention, showed that the overall parental stressor scale score decreased two weeks after the mindfulness intervention [8]. Both are small studies of 55 dyads and 27 mothers, respectively.

Marshall et al. showed no difference from enrollment to discharge in parental stress after a mindfulness-based training session [6]. This group provided only one education session to the parents, which occurred away from the bedside and did not include a control group. We believe that providing coaching at the bedside led to the increased acceptability of our intervention, with 30 of the 40 mothers who were approached giving consent and 14 of 15 MKC mothers reporting the intervention as "acceptable." Marshall reported a 51% consent rate, but only 29% of those approached completed the mindfulness training.

Parental stress in the NICU is subject to many factors. Stress may be related to the degree of prematurity. In this study, both groups had similar mean gestational age and included babies at the margin of viability. Parental stress tends to fluctuate as the baby's clinical condition improves or deteriorates over time. In this study, both groups were enrolled when the babies were approximately two weeks of age. Using the PSS at two points in time may not measure fluctuating stress due to the "roller-coaster" of stress resulting from rapid, unexpected changes in the babies' clinical condition. Stress could be measured more often (e.g. weekly) and for a longer duration to more accurately document the effect of mindfulness on parental stress throughout the NICU stay.

Neither anxiety nor depression scores were different between groups, but a statistically significant reduction of both anxiety and depression scores occurred over time for the MKC group. Similar results were found by Mendlesohn et al., who evaluated anxiety and depression scores using different scales and showed a within-group significant reduction but no between-group difference [8].

The mindfulness exercises, which were created for this study, resulted in a significant increase in both mindful awareness scores of curiosity and decentering when compared to the control group. Two other studies that measured mindfulness did not show any within-group variation in their mindfulness scores [6, 8]. It is possible that the difference is a reflection of the mindfulness scale that was used to measure mindful awareness. The MKC study offered ongoing coaching on a face-to-face basis, whereas the other two studies offered an initial mindfulness training session and encouraged the participants to practice. All studies provided MP3 audio recordings [6, 8]. It is also possible that the ongoing mindfulness coaching at the bedside was a key element that resulted in improving the mindful awareness scores and should be considered in the design of future studies.

Prior experience with mindfulness was reported by just under half the mothers in each group. The number of KC sessions and time per session were similar for both groups. This suggests that the positive results for the MKC mothers occurred as a result of the study intervention and were not simply related to standard kangaroo care or mothers' prior mindfulness practice. Kangaroo Care itself has been shown to reduce stress [13]. Mindfulness is also known to reduce stress [5]. Therefore, it is reasonable to assume that the combination might have an additive effect on the reduction of stress, which our pilot study has now demonstrated. A more extensive study is required to explore the synergistic effects of these two interventions; and whether they are additive or multiplicative.

This study demonstrated the feasibility of MKC in a level 3 NICU. More than half of the mothers gave birth between 23 and 27 weeks of gestation. The mindfulness intervention was conducted during KC for babies who were ventilated, including some with high-frequency oscillatory ventilation, and during KC for recently extubated babies who were on non-invasive mechanical ventilation.

The primary cost of the intervention was time. The median duration was 30 min for the first two visits, in which approximately half of the visit was centred on practicing the mindfulness exercise. During the third- and fourth-week encounters, most mothers in the MKC group verbally expressed that they had a good grasp of both mindfulness techniques. Thus, the median duration of those last two visits was 7 min. However, this does not include additional time taken to coordinate best times with mothers for these meetings and rescheduling meetings when mothers could not do so at the expected times. This intervention would likely need a dedicated member of staff who can provide the coaching, at times convenient to the mothers.

## Conclusion

This study is an attempt to bring mindfulness to the bedside at a moment when NICU mothers are generally available. This is the first study to show significant within-group reductions in maternal stress, anxiety and depression symptoms and a significant between-group increase of mindful awareness in mothers of premature babies who were coached in and practiced two simple mindfulness exercises during KC. This study also demonstrated the feasibility and the acceptability of MKC in the NICU. These findings need to be replicated in a more extensive study to establish generalisability. Further study could explore different mindfulness techniques and tailoring mindfulness exercises to mothers' individual needs.

## Supplementary Information

**Additional file 1**. Baby Body Scan, Instructions. Written script of the audio file for the first encounter’s mindfulness technique in which parents are invited to develop the awareness of any sensations felt in the body while holding their baby in KC, with an emphasis on the physical sensations created by the presence of their baby. This file includes the instructions.

**Additional file 2**. Baby Body Scan, Guided Practice. Written script of the audio file for the first encounter’s mindfulness technique in which parents are invited to develop the awareness of any sensations felt in the body while holding their baby in KC, with an emphasis on the physical sensations created by the presence of their baby. This file includes the guided practice.

**Additional file 3**. Nurture Positive Feelings While Holding Baby, Instructions. Written script of the audio file for the second encounter’s mindfulness technique in which parents are invited to create positive emotional sensations in the body triggered by holding their baby skin-to-skin. This file includes the instructions.

**Additional file 4**. Nurture Positive Feelings While Holding Baby, Guided Practice. Written script of the audio file for the second encounter’s mindfulness technique in which parents are invited to create positive emotional sensations in the body triggered by holding their baby skin-to-skin. This file includes the guided practice.

**Additional file 5**. Maternal Demographic Form. This is a general demographic form that all mothers were asked to complete at enrolment.

**Additional file 6**. Kangaroo Care Log Intervention Group. Log on which mothers were asked to record the timing and duration of each KC session and document concurrent activities during KC.

**Additional file 7**. Kangaroo Care Log Control Group. Log on which mothers were asked to record the timing and duration of each KC session and document concurrent activities during KC.

**Additional file 8**. Acceptability form. 3-point Likert scale created by the research team to measure the acceptability of the different instruments and mindfulness intervention completed by the mothers at the end of the study.

**Additional file 9**. Feasibility form. Tool created by the research team, filled out during the study for every mother recruited after each step of the research project with the following components: recruitment capability, data collection procedures and outcome measures, resources and ability to implement the study.

## Data Availability

The datasets used and analyzed during the current study are available from the corresponding author on reasonable request.

## Abbreviations

KC: Kangaroo Care
MKC: Mindful Kangaroo Care
NICU: Newborn Intensive Care Unit
SC: Standard of Care
