# Supplementary material for: Mindful Kangaroo Care: mindfulness intervention for mothers during skin-to-skin care: a randomized control pilot study
Source: BMC Pregnancy Childbirth. 2022 Jan 15;22:35. doi: 10.1186/s12884-021-04336-w (PMC8761274; doi:10.1186/s12884-021-04336-w)
Supplement: Supplementary file 1 — Additional file 1. Baby Body Scan, Instructions. Written script of the audio file for the first encounter’s mindfulness technique in which parents are invited to develop the awareness of any sensations felt in the body while holding their baby in KC, with an emphasis on the physical sensations created by the presence of their baby. This file includes the instructions. [file 12884_2021_4336_MOESM1_ESM.pdf]

Mindful Kangaroo Care: Mindfulness Intervention for mothers during skin-to-skin care: a randomized control pilot study.

Additional file #1: Baby body scan, instructions

Author: Marc-Antoine Landry

What

During this first mindfulness exercise, the goal is to simply acknowledge or notice the presence of any sensations felt in your body while holding your baby.

It could be a physical sensation such as pressure, tension, pain, warmth, cold, vibration, contact of your body with your baby, feeling some clothes touching your skin, a breeze on your skin or even something that you don't really know what it is, but you feel it with your body.

It could also be an emotional sensation like love, joy, surprise, anger, sadness, fear, which you may feel in the throat, belly, shoulders or chest, for instance.

Why

Body sensations keep us anchored in the present moment. This mindfulness practice focuses away from thinking of the non-present content by constantly bringing back the awareness to body sensations as they occur.

This mindfulness exercise cultivates an open and accepting attitude toward any experience with a curious, detached, and non-reactive mind. Judgments and reactions arise constantly and naturally. This mindfulness practice focuses away from the judgments and mental reactions and allows not getting caught into the cycle of judging the judging and thus helps to develop a non-judgmental attitude toward one's experiences.

When/Where

I would encourage you to practice this mindfulness technique every time you hold your baby in the NICU, more specifically during kangaroo care.

Watch out

Physical and emotional sensations may be subtle to detect for some people. Subtle is enough to work within a mindfulness practice. There is no need to have a particular kind of experience. You just need to focus on whatever you happen to notice and let go of any expectations or judgments.

Physical and emotional sensations can be strong and overwhelming for some people. There are at least two mindful ways to deal with this situation. The first one is to turn toward the sensation and deconstruct the sensation in terms of location, quality, intensity, how it is changing or how it is interacting with other sensations. By becoming curious and granular in the details, it may make the unpleasant sensation lose its grip. Another mindful strategy would be to turn away from the unpleasant sensation where you would allow the sensation to be in the background, and you would not focus on it. You would rather focus on other sensations felt elsewhere in the body.

And another common issue is being distracted from the task. In this exercise, we are only noticing the sensations felt in the body. Any auditory or visual sensory events, whether in your mind, like mental talk and mental images, or outside of your mind like physical sound (alarms, conversations) and physical sight (people, objects), would be a form of distraction. Every time your attention is drawn to such a distraction, you are asked to kindly bring your attention into your body and notice what sensation you are experiencing in the body at that moment. The auditory or visual distractions may still be there, but you would leave them in the background as you focus on the sensations felt in the body.

How

First, you will bring your attention to a place in your body that is in physical contact with your baby. For example, you could bring your attention to your hands that are holding your baby or to your chest that is in close contact with your baby. We'll explore that for a minute or two.

Then, you will be asked to detect and explore the sensations you feel inside your body when you place your attention on the area in physical contact with your baby's head. Slowly, you will move your attention from the place where you feel your baby's head to the place where you feel your baby's torso. Again you will detect and explore the sensations that you feel inside your body when you place your attention on that new point of contact with your baby. And lastly, you will repeat the same exploration when you place your attention where you feel your baby's limbs. You will do this a few times, slowly, moving from the head of your baby to your baby's toes and back from your baby's toes to your baby's head.

When you move your attention over all those different places, the main instruction is to notice the presence of any sensations in your body, whether it is a physical sensation or an emotional sensation. You should take your time to explore the sensations that you detect in your body. Once you have noticed a sensation, you may want to stay connected to that sensation for a brief instant, like a few seconds or so.

After noticing the sensation, you may want to open up to that sensation and get curious about it. You may want to discover where it is, how much of it there is and if it is changing in any way. You don't need to answer any of those questions in your mind. You actually only need to notice the sensation in your awareness.

The sensation does not need to be experienced at the point of contact with your baby; it could be experienced in another location in your body. For example, after bringing your attention to your baby's legs, it may trigger a pleasant, loving feeling in your chest. First, you may want to explore and stay connected to the physical sensation of holding the leg in your hand for few seconds, and then you may want to explore the emotional sensation of love that you feel in your chest for another few seconds. And then, you would explore other or the same sensations triggered by bringing your attention to your baby's legs.
