# Supplementary material for: Mindful Kangaroo Care: mindfulness intervention for mothers during skin-to-skin care: a randomized control pilot study
Source: BMC Pregnancy Childbirth. 2022 Jan 15;22:35. doi: 10.1186/s12884-021-04336-w (PMC8761274; doi:10.1186/s12884-021-04336-w)
Supplement: Supplementary file 2 — Additional file 2. Baby Body Scan, Guided Practice. Written script of the audio file for the first encounter’s mindfulness technique in which parents are invited to develop the awareness of any sensations felt in the body while holding their baby in KC, with an emphasis on the physical sensations created by the presence of their baby. This file includes the guided practice. [file 12884_2021_4336_MOESM2_ESM.pdf]

Mindful Kangaroo Care: Mindfulness Intervention for mothers during skin-to-skin care: a randomized control pilot study.

Additional file #2: Baby body scan, guided practice

Author: Marc-Antoine Landry

Audio version available here: https://youtu.be/2KykcTyIcv8

"Baby body scan mindfulness guided practice

"This is a mindfulness practice to discover and explore body sensations while holding your baby in skin to skin contact or kangaroo care.

Posture Instructions (60+ seconds)

> "I invite you to take a deep breath
> "And then relax into a comfortable posture.
> "Where you are relaxed but yet alert.
> "You may want to drop your jaw, drop your shoulders
> "You may want to close your eyes or leave them open as your prefer
> "And now, bring your awareness into the body

General exploration of sensations while holding the baby (3 min)

> "Whenever you're ready, bring your attention where your body touches your baby (pause)
> "Allow your attention to detect, in your body, any physical or emotional sensations
>
> "Maybe you feel the warmth of your baby's body, your baby's breathing movements, or that your baby is moving against your chest.
> "Maybe you feel the lightness or the heaviness of your baby in your arms
> "Maybe you feel the relaxation in your body while holding your baby or your feel area of discomfort or some tension while holding your baby.
> "Maybe you feel love, tenderness, joy, pleasure, amazement, contentment, hope.
> "Maybe you feel worry, fear, anxiety, sadness, displeasure, guilt, agitation, frustration.
>
> "Any of that is fine. Just notice whatever is present in your current experience.
>
> "When you have noticed a sensation,
> stay connected to that sensation for a brief instant, a few seconds or so
> "Open up to that sensation and get curious about it.
> "You may want to discover with you awareness where is the sensation, how much of it ou feel or if the sensation is changing in any way.

“There is no need to describe or answer any of those questions in your mind.
“You only need to notice the sensation and those details with your awareness.

“Then, allow your attention to detect another or the same body sensation.
“And soak into that experience for few seconds
“And repeat the cycle a few more times.

“At any time, you may want to bring back your attention where your feel your baby to trigger some physical or emotional sensations in your body.

Baby body scan (10 min)

“Now, we will start exploring the sensations that are present when you bring your attention to different parts of your baby’s body that are in contact with you.

“I invite you to explore the sensations associated with the contact of your **baby’s head** (3 min)

“Bring your attention to the area where you are in physical contact with your baby’s head
“Get curious about the sensations that you feel around your baby’s head.
“Stay connected to those sensations for a brief instant, a few seconds or so
“And then, explore other or the same sensations that are triggered by bringing your attention to your baby’s head

“Great, now

“I invite you to bring your attention to the area where you are in contact of your **baby’s chest and/or back** (2 min)

“Explore and be curious about the sensations that you feel around your baby’s torso
“Stay connected to those sensations for a brief instant, a few seconds or so
“And keep exploring and noticing your sensations.

“Remember that the sensations that you feel do not need to be only experienced at the point of contact with your baby; it could be experienced elsewhere in your body
“But, for the moment, we are only interested in the sensations that are directly related or remotely triggered by being in contact with your baby’s chest and/or back

“Great, now

“I invite you to bring your attention to the area where you are in contact with your **baby’s arms and/or legs** (2 min)

“Discover the sensations that you feel when in contact with your baby’s limbs
“Stay connected to those sensations for a brief instant, a few seconds or so

“And then explore other or the same sensations that are triggered by bringing your attention to your baby’s limbs

“Great, now

“I invite you to bring back your attention to the area where you are in contact of your **baby’s torso, whether it’s your baby’s chest and/or back** (2 min)

“Get curious about the sensations that you feel
“Stay connected to those sensations for a brief instant
“And then explore other or the same sensations

“Remember that the sensation that you feel do not need to be only experienced at the point of contact with your baby’s torso; it could be experienced elsewhere in your body
“But, for the moment, we are only interested in the sensations that are directly related or remotely triggered by being in contact with your baby’s chest and/or back

“Great, now

“I invite you to bring your attention to where you feel your **baby’s head** (2 min)

“Get curious about the sensations that you feel
“Stay connected to those sensations for a brief instant, a few seconds or so
“And then explore other or the same sensations

Final exploration of sensations while holding baby as a whole (2 min)

“Great, now

“Bring your attention to your baby as a whole; head, torso and limbs all connected. (pause)
“Allow your attention to detect any physical or emotional sensations that you may feel in your body.

“When you have noticed a sensation,
stay connected to that sensation for a brief instant

“Open up to that sensation and get curious about it.

“Then, allow your attention to detect another sensation.

“And repeat this cycle a few more times.

“At any time, you may want to bring back your attention where your feel your baby to trigger some more physical or emotional sensations in your body.

At 16 min (End)

“OK. Good work, well done
