# Supplementary material for: Mindful Kangaroo Care: mindfulness intervention for mothers during skin-to-skin care: a randomized control pilot study
Source: BMC Pregnancy Childbirth. 2022 Jan 15;22:35. doi: 10.1186/s12884-021-04336-w (PMC8761274; doi:10.1186/s12884-021-04336-w)
Supplement: Supplementary file 3 — Additional file 3. Nurture Positive Feelings While Holding Baby, Instructions. Written script of the audio file for the second encounter’s mindfulness technique in which parents are invited to create positive emotional sensations in the body triggered by holding their baby skin-to-skin. This file includes the instructions. [file 12884_2021_4336_MOESM3_ESM.pdf]

Mindful Kangaroo Care: Mindfulness Intervention for mothers during skin-to-skin care: a randomized control pilot study.

Additional file #3: Nurture positive feelings while holding baby, instructions

Author: Marc-Antoine Landry

What

During this second mindfulness exercise, the goal is to create positive feelings in the body while holding your baby. By positive sensations, we mean anything that you can experience in the body, such as love, tenderness, joy, pleasure, amazement, contentment or hope to name some examples. Typically, those sensations are felt in the chest, but some people may also experience them in the face, throat, upper belly, or other body parts.

Why

From a mindfulness perspective, body sensations keep us anchored in the present. Similar to the first exercise, this mindfulness practice focuses away from the non-present content (the thinking mind) by constantly bringing back the awareness to the positive sensations in the body.

Similar to the first exercise, this mindfulness exercise cultivates an open and accepting attitude toward the experience felt in the body. This mindfulness practice also focuses away from the judgments and the mental reactions and allows not to get caught into the cycle of judging the judging and thus helps to develop a non-judgmental attitude toward one's experiences.

From an attachment perspective, giving birth prematurely creates a separation between you and your baby. Re-establishing an emotional connection between you and your baby seems to play a key role in the recovery process. This mindfulness exercise specifically fosters positive emotions that you can create while holding your baby to re-establish or nurture that connection.

When/Where

I would encourage you to practice this mindfulness technique every time you hold your baby in the NICU, more specifically during skin-to-skin care or kangaroo care.

Watch out

Sometimes, positive emotional sensations may be very subtle to detect in the body. Detecting emotions in the body is a new skill for some, and in our culture, we typically haven't been trained to do that. It's OK to search and guess while developing this skill. Remember that subtle is enough to work within a mindfulness practice. There is no need to have a particular kind of experience. You just need to focus on whatever you happen to notice and let go of any expectations or judgments.

Sometimes when you try to focus on the positive, the negative may come instead or may come at the same time. This is not uncommon, and there is nothing wrong with you. For some people, this can be quite disturbing. If you are able to concentrate on subtle positive emotions while strong negative emotions are present in the body, this will strengthen your positivity muscle. If today you are unable to do this, you are always free to switch to our first technique and try this new one another time.

Sometimes, creating positivity may seem inauthentic. You may be concerned that the emotion is not genuine, or you are trying to suppress the true feelings. You are actually not asked to suppress any emotions. Whatever would come up that is not positive, you would allow it to come, be and go in the background while you maintain your focus on the positive sensations in the body that you are creating. Remember that you are working the muscle of positivity, and like in any training, it may not work the first time you try it, or it may feel awkward the first time you try it.

And, as in many mindfulness techniques, another comment issue is being distracted from the task. Any auditory or visual sensory events, whether internal like mental talk and mental images or external like physical sound (alarm, conversations) and physical sight (people, objects), would be a form of distraction. In this case, physical body sensations like pain, tension or discomfort would also be considered as distractions. Every time your attention is drawn to such a distraction, you are asked to kindly bring your attention into your body where you experience the positive feelings and/or re-generate positive feelings in your body. The distraction may still be there, but you would leave it in the background as you are focusing on the positive sensations felt in the body.

How

The main instruction for this practice is to create and pay attention to positive feelings in the body. After you have found a way to activate positive emotional sensations in the body, you bring the awareness where you feel that sensation in your body. Then, you focus on the pleasant sensation, get curious about it, open up to it until it fades. When it’s gone, you reactivate the positive emotional sensation as if you were striking the bell of positivity in your body. You stay connected to that sensation until it vanishes, and you repeat these steps over and over.

You are not asked to determine or name what type of emotional sensation you are experiencing, but only to notice the sensation in the body. You may want to discover where it is, how much of it there is and if it is changing in any way. You don’t need to answer any of those questions in your mind. You only want to notice those details with your awareness.

While you are holding your baby in a skin-to-skin position, a series of different triggers like feeling the contact of your baby on your skin, feeling your baby’s breathing movements, looking at your baby, smelling your baby, or thinking positively about your baby will be offered during

the guided practice to explore how these different experiences can help you create positive sensations in your body. There is no need to have a particular kind of experience. You just need to focus on the positive sensation you happen to notice until it fades and then reactivate another one. And you do this over and over.
