# Supplementary material for: Mindful Kangaroo Care: mindfulness intervention for mothers during skin-to-skin care: a randomized control pilot study
Source: BMC Pregnancy Childbirth. 2022 Jan 15;22:35. doi: 10.1186/s12884-021-04336-w (PMC8761274; doi:10.1186/s12884-021-04336-w)
Supplement: Supplementary file 4 — Additional file 4. Nurture Positive Feelings While Holding Baby, Guided Practice. Written script of the audio file for the second encounter’s mindfulness technique in which parents are invited to create positive emotional sensations in the body triggered by holding their baby skin-to-skin. This file includes the guided practice. [file 12884_2021_4336_MOESM4_ESM.pdf]

Mindful Kangaroo Care: Mindfulness Intervention for mothers during skin-to-skin care: a randomized control pilot study.

Additional file #4: Nurture positive feelings while holding baby, guided practice

Author: Marc-Antoine Landry

Audio version available here: https://youtu.be/I0LHBZS5Eps

"Nurture positive feelings while holding baby, mindfulness guided practice

"This is a mindfulness practice to create, discover and explore positive emotional sensations felt in the body while holding your baby in skin-to-skin contact or kangaroo care.

Posture Instruction (60+ seconds)

"I invite you to take a deep breath
"And then relax into a comfortable posture.
"Where you are relaxed but yet alert.
"You may want to drop your jaw, drop your shoulders
"You may want to close your eyes or leave them open as your prefer
"And now, bring your awareness into the body

Initial Instruction + exploration (3 min)

"Whenever you're ready,
"See if just being with your baby naturally brings a positive emotional sensation in your body

"For example, the positive emotional sensation could be love, tenderness, joy, pleasure, amazement, contentment or hope to name a few.

"If you detect a positive sensation, bring your awareness where you detect it
"And, gently stay connected to that sensation until it vanishes

"You do not need to determine or name what type of emotional sensation you are experiencing, but only notice it with your awareness

"Be curious about it.
"You may want to discover where it is, how much of it there is or if it is changing in any way
"You actually don't need to answer any of those questions in your mind
"You only need to notice the sensations with your awareness

“*When the positive sensation vanishes,*
“See if just being with your baby naturally brings another positive feeling in your body
“If so, bring your awareness where you detect the positive sensation in your body and
“Stay connected to the sensation again until it vanishes
“And repeat this process a few more times.

“If you don’t feel any positive emotional sensations at this time, just maintain your attention on your baby.
“We will explore other ways to trigger positive feelings.

Trigger 1*: feeling the contact of your baby on your skin* (3 min)

“Now, I invite you to explore the positive emotional sensations that you may experience in your body when feeling your baby on your skin.

“Take a moment to feel the contact where your body and your baby’s body meet. Maybe you feel some warmth or the breathing movements of your baby. Maybe you feel his or her presence on you.

(pause few seconds)

“Now, notice if that physical contact with your baby created a positive emotional sensation in your body.
“It can be subtle, but subtle is enough to work with.

“You don’t need to describe what you feel; just *notice the sensation*
“Open up to the sensation that you are experiencing
“Be curious about it.
“And stay connected to it until it vanishes

“*When the pleasant sensation is gone,*
“Bring back your attention to where your feel your baby’s body.
“Or where you feel your baby’s breathing movements against your body.

“See if that physical contact with your baby brings another positive feeling in your body
“If so, bring your awareness where you detect the positive sensation and
“Stay connected to the sensation until it vanishes

(pause)

“And repeat this process of feeling your baby against you to create a positive emotional sensation and focus on the sensation in the body until it vanished.
“If you don’t feel any positive emotional sensation at this time, just maintain your attention on your baby. We will explore other ways to trigger positive feelings.

“Great, let go of that.

Trigger 2*: looking at your baby* (3 min)

“Now, I invite you to explore the positive emotional sensations that you may feel in your body when looking at your baby.

“You may look at your baby’s head, face, body, hands or whatever you can see from your perspective. You don’t need to move your baby.

(pause 10 seconds)

“If looking at your baby triggers a positive emotional sensation, bring back your awareness in your body and focus on that sensation as we did before

“There is no need to have a particular kind of experience

“Just notice whatever is present in your current experience.

“Open up to the sensation that you are experiencing

“Be curious about it and

“Stay connected to it until it vanishes

“*When the positive sensation is gone,*

“Bring back your attention to looking at your baby

“See, by simply looking at your baby re-strikes the bell of positive feelings in your body

“If so, bring your awareness in your body where you detect that positive sensation and

“Stay connected to the sensation until it vanishes

(pause)

“And repeat this process of looking at your baby to create a positive sensation and focus on the sensation in the body until it vanished over and over.

“Great, let go of that.

Trigger 3*: pleasant visualization about your baby* (3 min)

“Now, I invite you to explore the positive emotional sensations that you may feel in your body when you create a positive image of your baby.

“I invite you to create an image in your mind, with your eyes closed -- an image about your baby that would trigger positive emotions. It can be an image about the future that you dream of, an image about a place or people that you would see your baby with. Or it could be an image of something that has already happened. An image of your baby that would make you feel good.

“The image of your baby that you create in your mind can be vague or fleeting
It does not matter, as the goal is to actually create positive emotional sensations in the body with whatever image that you can create in your mind
(pause 10 seconds)
“When you notice a positive sensation in your body, let go of the image that you created in your mind and
“Bring your awareness into your body where you notice that positive sensation

“Open up to that sensation, the sensation that you are experiencing
“Be curious about it and
“Stay connected to it until it vanishes

“*When the positive sensation is gone,*
“Bring another image or the same image in your mind to re-generate a positive sensation

“Again, when you notice a positive emotional sensation in your body, let go of the baby’s image in your mind and
“Bring your awareness into your body where you notice the positive sensation and
“Stay with it until it vanishes

(pause)

“And repeat this process over and over.

“Great, let go of that.

Trigger 4*: positive thought about your baby* (3 min)

“Now, I invite you to explore the positive emotional sensations that you may feel in your body when you have a positive thought about your baby.

“I invite you to say nice things to yourself, in your mind, about your baby. It could be a single word or a short phrase. Something lovely about your baby. A wish, a dream, a hope. Something you like to say to your baby. Something that brings a smile to your face. Something that reminds you how strong and beautiful your baby is. Anything that would trigger a positive emotion and make you feel good.
(pause 10 seconds)
“If you notice a positive emotional sensation in your body, let go of the words that you say to yourself in your mind and
“Bring your awareness into your body where you notice the positive sensation

“Open up to that positive emotional sensation that you experience in the body
“Be curious about it and
“Stay connected to it until it vanishes

“*When the positive emotional sensation is gone,*
“Say some more words to yourself about your baby to re-generate a positive emotional sensation
“It could be the same that you just said or something new

“When you notice a positive emotional sensation in your body, let go of the inner talk and
“Bring your awareness into your body where you notice that positive sensation and
“Stay with it until it vanishes

“And repeat this process over and over.

“Great, let go of that.

Trigger 5*: smelling your baby* (3 min)

“Now, I invite you to explore the positive emotional sensations that you may feel in your body when smelling your baby.

“Take a moment to smell your baby.
(pause 10 seconds)

“Whenever you notice the presence of a positive emotional sensation in your body, let go of the smell and bring back your awareness in your body where you feel that sensation

“Again, there is no need to have a particular kind of experience
“Just notice whatever is present in your current experience.

“Open up to that positive emotional sensation that you experience right now
“Be curious about it.
“Stay connected to it until it vanishes

“*When the positive emotional sensation is gone,*
“Smell your baby another time
“If smelling your baby triggers another positive emotional sensation in your body
“Bring your awareness in your body where you detect that positive sensation and
“Stay connected to it until it vanishes

“And repeat this process over and over.

“Great, let go of that.

Free exploration (3 min)

“Now, you can explore on your own to create positive emotional sensations in your body or focus on the positive emotional sensations that are already present in your body while holding your baby. Remember that you can create positive emotional sensations by feeling the contact of your baby on your skin, looking at your baby, creating a positive image of your baby in your mind, saying nice things about your baby in your mind or smelling your baby.

“You can use any of those ideas or mix some ideas together.
“When you have created a positive emotional sensation in your body,
“Bring your full awareness in your body and
“Focus on that positive sensation until it vanishes
“Then re-strike the bell of positivity and repeat these steps over and over

“OK. Great work, well done
You may carry that positivity as you transition to your other task today
(end)
