# Supplementary material for: Mindful Kangaroo Care: mindfulness intervention for mothers during skin-to-skin care: a randomized control pilot study
Source: BMC Pregnancy Childbirth. 2022 Jan 15;22:35. doi: 10.1186/s12884-021-04336-w (PMC8761274; doi:10.1186/s12884-021-04336-w)
Supplement: Supplementary file 5 — Additional file 5. Maternal Demographic Form. This is a general demographic form that all mothers were asked to complete at enrolment. [file 12884_2021_4336_MOESM5_ESM.pdf]

Study # ____

Mindful Kangaroo Care: Mindfulness Intervention for mothers during skin-to-skin care: a randomized control pilot study.

Additional file #5: Maternal Demographic Form

A- What is your age: _______ years

B- What is the highest degree or level of school you have completed? If currently enrolled, highest degree received?

1- Less than high school
2- High school degree
3- College or Undergraduate degree
4- Graduate degree

C) How many pregnancies did you have including this last one and including any losses ? ____

D) How many babies did you deliver (term or preterm) including this last one? _____

E) Were your other children born premature (before 37 weeks)? Yes / No / not applicable

F) Were your other children hospitalized in Newborn Intensive Care Unit (NICU)?
Yes / No / not applicable

G) Do you have any prior experience with holding skin-to-skin with your other children?
Yes / No / not applicable

H) Was your prior experience with kangaroo care or holding skin-to-skin in an NICU?
Yes / No / not applicable

I) Do you practice mindfulness? Yes / No
If yes, for how long (in years have you been practicing): ________

J) Have you ever had a diagnosis of depression? Yes / No

K) Have you ever had a diagnosis of anxiety? Yes / No

L) Gestational Age of this baby: _______ weeks + ___/7 days

M) Birth weight of this baby: ________ grams

N) Delivery type: Vaginal / Cesarean

O) At what age this current baby and you had your first skin-to-skin experience (in days)? _____

For researchers only

At what age we start the data collection (in days)? _____
