# Supplementary material for: Mindful Kangaroo Care: mindfulness intervention for mothers during skin-to-skin care: a randomized control pilot study
Source: BMC Pregnancy Childbirth. 2022 Jan 15;22:35. doi: 10.1186/s12884-021-04336-w (PMC8761274; doi:10.1186/s12884-021-04336-w)
Supplement: Supplementary file 6 — Additional file 6. Kangaroo Care Log Intervention Group. Log on which mothers were asked to record the timing and duration of each KC session and document concurrent activities during KC. [file 12884_2021_4336_MOESM6_ESM.pdf]

Study # ____

Mindful Kangaroo Care: Mindfulness Intervention for mothers during skin-to-skin care: a randomized control pilot study.
Additional file #6: Kangaroo Care Log Intervention Group

Week 1:

Please write date and circle D for day time (7am-4pm), E for evening (after 4pm)
Please write approximately how long in minutes lasted the kangaroo care

Circle approximately how much time you spent 1- practicing mindfulness, 2- using your smartphone, 3- sleeping and 4-engaged in a conversation with people around you during kangaroo care (0% - 25% - 50% - 75% or 100%)

Last question (How satisfied ...): Circle 1= Very dissatisfied, 2= Slightly dissatisfied, 3= Neutral, 4= Slightly Satisfied, 5= Very satisfied

Date & Time (Month/Day) Day/Evening	Kangaroo Care Duration in minutes (approx)	Mindfulness exercise for how much time? From 0-100%	Smartphone for how much time? From 0-100%	Sleeping for how much time? From 0-100%	Conversation for how much time? From 0-100%	How satisfied were you after this Kangaroo care?
____/____ D/E	minutes	0/ 25/ 50/ 75/ 100	0/ 25/ 50/ 75/ 100	0/ 25/ 50/ 75/ 100	0/ 25/ 50/ 75/ 100	1 / 2 / 3 / 4 / 5
____/____ D/E	minutes	0/ 25/ 50/ 75/ 100	0/ 25/ 50/ 75/ 100	0/ 25/ 50/ 75/ 100	0/ 25/ 50/ 75/ 100	1 / 2 / 3 / 4 / 5
____/____ D/E	minutes	0/ 25/ 50/ 75/ 100	0/ 25/ 50/ 75/ 100	0/ 25/ 50/ 75/ 100	0/ 25/ 50/ 75/ 100	1 / 2 / 3 / 4 / 5
____/____ D/E	minutes	0/ 25/ 50/ 75/ 100	0/ 25/ 50/ 75/ 100	0/ 25/ 50/ 75/ 100	0/ 25/ 50/ 75/ 100	1 / 2 / 3 / 4 / 5
____/____ D/E	minutes	0/ 25/ 50/ 75/ 100	0/ 25/ 50/ 75/ 100	0/ 25/ 50/ 75/ 100	0/ 25/ 50/ 75/ 100	1 / 2 / 3 / 4 / 5
____/____ D/E	minutes	0/ 25/ 50/ 75/ 100	0/ 25/ 50/ 75/ 100	0/ 25/ 50/ 75/ 100	0/ 25/ 50/ 75/ 100	1 / 2 / 3 / 4 / 5
____/____ D/E	minutes	0/ 25/ 50/ 75/ 100	0/ 25/ 50/ 75/ 100	0/ 25/ 50/ 75/ 100	0/ 25/ 50/ 75/ 100	1 / 2 / 3 / 4 / 5
____/____ D/E	minutes	0/ 25/ 50/ 75/ 100	0/ 25/ 50/ 75/ 100	0/ 25/ 50/ 75/ 100	0/ 25/ 50/ 75/ 100	1 / 2 / 3 / 4 / 5

Have you listened to the mindfulness instruction audio recording this week? Yes / No
Have you listened to the mindfulness guided practice audio recording this week? Yes / No
Have you practiced any other mindfulness while not offering skin-to-skin care? Yes / No (if yes please detail below)

Any comments to help this study or about your mindfulness experience:

____
____
____

Study # ____

Mindful Kangaroo Care: Mindfulness Intervention for mothers during skin-to-skin care: a randomized control pilot study.
Additional file #6: Kangaroo Care Log Intervention Group

Week 2:

Please write date and circle D for day time (7am-4pm), E for evening (after 4pm)
Please write approximately how long in minutes lasted the kangaroo care

Circle approximately how much time you spent 1- practicing mindfulness, 2- using your smartphone, 3- sleeping and 4-engaged in a conversation with people around you during kangaroo care (0% - 25% - 50% - 75% or 100%)

Last question (How satisfied ...): Circle 1= Very dissatisfied, 2= Slightly dissatisfied, 3= Neutral, 4= Slightly Satisfied, 5= Very satisfied

Date & Time (Month/Day) Day/Evening	Kangaroo Care Duration in minutes (approx)	Mindfulness exercise for how much time? From 0-100%	Smartphone for how much time? From 0-100%	Sleeping for how much time? From 0-100%	Conversation for how much time? From 0-100%	How satisfied were you after this Kangaroo care?
____/____ D/E	minutes	0/ 25/ 50/ 75/ 100	0/ 25/ 50/ 75/ 100	0/ 25/ 50/ 75/ 100	0/ 25/ 50/ 75/ 100	1 / 2 / 3 / 4 / 5
____/____ D/E	minutes	0/ 25/ 50/ 75/ 100	0/ 25/ 50/ 75/ 100	0/ 25/ 50/ 75/ 100	0/ 25/ 50/ 75/ 100	1 / 2 / 3 / 4 / 5
____/____ D/E	minutes	0/ 25/ 50/ 75/ 100	0/ 25/ 50/ 75/ 100	0/ 25/ 50/ 75/ 100	0/ 25/ 50/ 75/ 100	1 / 2 / 3 / 4 / 5
____/____ D/E	minutes	0/ 25/ 50/ 75/ 100	0/ 25/ 50/ 75/ 100	0/ 25/ 50/ 75/ 100	0/ 25/ 50/ 75/ 100	1 / 2 / 3 / 4 / 5
____/____ D/E	minutes	0/ 25/ 50/ 75/ 100	0/ 25/ 50/ 75/ 100	0/ 25/ 50/ 75/ 100	0/ 25/ 50/ 75/ 100	1 / 2 / 3 / 4 / 5
____/____ D/E	minutes	0/ 25/ 50/ 75/ 100	0/ 25/ 50/ 75/ 100	0/ 25/ 50/ 75/ 100	0/ 25/ 50/ 75/ 100	1 / 2 / 3 / 4 / 5
____/____ D/E	minutes	0/ 25/ 50/ 75/ 100	0/ 25/ 50/ 75/ 100	0/ 25/ 50/ 75/ 100	0/ 25/ 50/ 75/ 100	1 / 2 / 3 / 4 / 5
____/____ D/E	minutes	0/ 25/ 50/ 75/ 100	0/ 25/ 50/ 75/ 100	0/ 25/ 50/ 75/ 100	0/ 25/ 50/ 75/ 100	1 / 2 / 3 / 4 / 5

Have you listened to the mindfulness instruction audio recording this week? Yes / No
Have you listened to the mindfulness guided practice audio recording this week? Yes / No
Have you practiced any other mindfulness while not offering skin-to-skin care? Yes / No (if yes please detail below)

Any comments to help this study or about your mindfulness experience:

____

____

____

Study # ____

Mindful Kangaroo Care: Mindfulness Intervention for mothers during skin-to-skin care: a randomized control pilot study.
Additional file #6: Kangaroo Care Log Intervention Group

Week 3:

Please write date and circle D for day time (7am-4pm), E for evening (after 4pm)
Please write approximately how long in minutes lasted the kangaroo care

Circle approximately how much time you spent 1- practicing mindfulness, 2- using your smartphone, 3- sleeping and 4-engaged in a conversation with people around you during kangaroo care (0% - 25% - 50% - 75% or 100%)

Last question (How satisfied ...): Circle 1= Very dissatisfied, 2= Slightly dissatisfied, 3= Neutral, 4= Slightly Satisfied, 5= Very satisfied

Date & Time (Month/Day) Day/Evening	Kangaroo Care Duration in minutes (approx)	Mindfulness exercise for how much time? From 0-100%	Smartphone for how much time? From 0-100%	Sleeping for how much time? From 0-100%	Conversation for how much time? From 0-100%	How satisfied were you after this Kangaroo care?
____/____ D/E	minutes	0/ 25/ 50/ 75/ 100	0/ 25/ 50/ 75/ 100	0/ 25/ 50/ 75/ 100	0/ 25/ 50/ 75/ 100	1 / 2 / 3 / 4 / 5
____/____ D/E	minutes	0/ 25/ 50/ 75/ 100	0/ 25/ 50/ 75/ 100	0/ 25/ 50/ 75/ 100	0/ 25/ 50/ 75/ 100	1 / 2 / 3 / 4 / 5
____/____ D/E	minutes	0/ 25/ 50/ 75/ 100	0/ 25/ 50/ 75/ 100	0/ 25/ 50/ 75/ 100	0/ 25/ 50/ 75/ 100	1 / 2 / 3 / 4 / 5
____/____ D/E	minutes	0/ 25/ 50/ 75/ 100	0/ 25/ 50/ 75/ 100	0/ 25/ 50/ 75/ 100	0/ 25/ 50/ 75/ 100	1 / 2 / 3 / 4 / 5
____/____ D/E	minutes	0/ 25/ 50/ 75/ 100	0/ 25/ 50/ 75/ 100	0/ 25/ 50/ 75/ 100	0/ 25/ 50/ 75/ 100	1 / 2 / 3 / 4 / 5
____/____ D/E	minutes	0/ 25/ 50/ 75/ 100	0/ 25/ 50/ 75/ 100	0/ 25/ 50/ 75/ 100	0/ 25/ 50/ 75/ 100	1 / 2 / 3 / 4 / 5
____/____ D/E	minutes	0/ 25/ 50/ 75/ 100	0/ 25/ 50/ 75/ 100	0/ 25/ 50/ 75/ 100	0/ 25/ 50/ 75/ 100	1 / 2 / 3 / 4 / 5
____/____ D/E	minutes	0/ 25/ 50/ 75/ 100	0/ 25/ 50/ 75/ 100	0/ 25/ 50/ 75/ 100	0/ 25/ 50/ 75/ 100	1 / 2 / 3 / 4 / 5

Have you listened to the mindfulness instruction audio recording this week? Yes / No
Have you listened to the mindfulness guided practice audio recording this week? Yes / No
Have you practiced any other mindfulness while not offering skin-to-skin care? Yes / No (if yes please detail below)

Any comments to help this study or about your mindfulness experience:

____

____

____

Study # ____

Mindful Kangaroo Care: Mindfulness Intervention for mothers during skin-to-skin care: a randomized control pilot study.
Additional file #6: Kangaroo Care Log Intervention Group

Week 4:

Please write date and circle D for day time (7am-4pm), E for evening (after 4pm)
Please write approximately how long in minutes lasted the kangaroo care

Circle approximately how much time you spent 1- practicing mindfulness, 2- using your smartphone, 3- sleeping and 4-engaged in a conversation with people around you during kangaroo care (0% - 25% - 50% - 75% or 100%)

Last question (How satisfied ...): Circle 1= Very dissatisfied, 2= Slightly dissatisfied, 3= Neutral, 4= Slightly Satisfied, 5= Very satisfied

Date & Time (Month/Day) Day/Evening	Kangaroo Care Duration in minutes (approx)	Mindfulness exercise for how much time? From 0-100%	Smartphone for how much time? From 0-100%	Sleeping for how much time? From 0-100%	Conversation for how much time? From 0-100%	How satisfied were you after this Kangaroo care?
____/____ D/E	minutes	0/ 25/ 50/ 75/ 100	0/ 25/ 50/ 75/ 100	0/ 25/ 50/ 75/ 100	0/ 25/ 50/ 75/ 100	1 / 2 / 3 / 4 / 5
____/____ D/E	minutes	0/ 25/ 50/ 75/ 100	0/ 25/ 50/ 75/ 100	0/ 25/ 50/ 75/ 100	0/ 25/ 50/ 75/ 100	1 / 2 / 3 / 4 / 5
____/____ D/E	minutes	0/ 25/ 50/ 75/ 100	0/ 25/ 50/ 75/ 100	0/ 25/ 50/ 75/ 100	0/ 25/ 50/ 75/ 100	1 / 2 / 3 / 4 / 5
____/____ D/E	minutes	0/ 25/ 50/ 75/ 100	0/ 25/ 50/ 75/ 100	0/ 25/ 50/ 75/ 100	0/ 25/ 50/ 75/ 100	1 / 2 / 3 / 4 / 5
____/____ D/E	minutes	0/ 25/ 50/ 75/ 100	0/ 25/ 50/ 75/ 100	0/ 25/ 50/ 75/ 100	0/ 25/ 50/ 75/ 100	1 / 2 / 3 / 4 / 5
____/____ D/E	minutes	0/ 25/ 50/ 75/ 100	0/ 25/ 50/ 75/ 100	0/ 25/ 50/ 75/ 100	0/ 25/ 50/ 75/ 100	1 / 2 / 3 / 4 / 5
____/____ D/E	minutes	0/ 25/ 50/ 75/ 100	0/ 25/ 50/ 75/ 100	0/ 25/ 50/ 75/ 100	0/ 25/ 50/ 75/ 100	1 / 2 / 3 / 4 / 5
____/____ D/E	minutes	0/ 25/ 50/ 75/ 100	0/ 25/ 50/ 75/ 100	0/ 25/ 50/ 75/ 100	0/ 25/ 50/ 75/ 100	1 / 2 / 3 / 4 / 5

Have you listened to the mindfulness instruction audio recording this week? Yes / No
Have you listened to the mindfulness guided practice audio recording this week? Yes / No
Have you practiced any other mindfulness while not offering skin-to-skin care? Yes / No (if yes please detail below)

Any comments to help this study or about your mindfulness experience:

____
____
____
