# Supplementary material for: Mindful Kangaroo Care: mindfulness intervention for mothers during skin-to-skin care: a randomized control pilot study
Source: BMC Pregnancy Childbirth. 2022 Jan 15;22:35. doi: 10.1186/s12884-021-04336-w (PMC8761274; doi:10.1186/s12884-021-04336-w)
Supplement: Supplementary file 7 — Additional file 7. Kangaroo Care Log Control Group. Log on which mothers were asked to record the timing and duration of each KC session and document concurrent activities during KC. [file 12884_2021_4336_MOESM7_ESM.pdf]

Study # ____

Mindful Kangaroo Care: Mindfulness Intervention for mothers during skin-to-skin care: a randomized control pilot study.
Additional file #7: Kangaroo Care Log Control Group

Week 1:

Please write date and circle D for day time (7am-4pm), E for evening (after 4pm)
Please write approximately how long in minutes lasted the kangaroo care

Circle approximately how much time you spent 1- using your smartphone, 2- sleeping and 3-engaged in a conversation with people around you during kangaroo care (0% - 25% - 50% - 75% or 100%)

Last question (How satisfied ...): Circle 1= Very dissatisfied, 2= Slightly dissatisfied, 3= Neutral, 4= Slightly Satisfied, 5= Very satisfied

Date & Time (Month/Day) Day/Evening	Kangaroo Care Duration in minutes (approx)	Smartphone for how much time? From 0-100%	Sleeping for how much time? From 0-100%	Conversation for how much time? From 0-100%	How satisfied were you after this Kangaroo care?
____/____ D/E	minutes	0/ 25/ 50/ 75/ 100	0/ 25/ 50/ 75/ 100	0/ 25/ 50/ 75/ 100	1 / 2 / 3 / 4 / 5
____/____ D/E	minutes	0/ 25/ 50/ 75/ 100	0/ 25/ 50/ 75/ 100	0/ 25/ 50/ 75/ 100	1 / 2 / 3 / 4 / 5
____/____ D/E	minutes	0/ 25/ 50/ 75/ 100	0/ 25/ 50/ 75/ 100	0/ 25/ 50/ 75/ 100	1 / 2 / 3 / 4 / 5
____/____ D/E	minutes	0/ 25/ 50/ 75/ 100	0/ 25/ 50/ 75/ 100	0/ 25/ 50/ 75/ 100	1 / 2 / 3 / 4 / 5
____/____ D/E	minutes	0/ 25/ 50/ 75/ 100	0/ 25/ 50/ 75/ 100	0/ 25/ 50/ 75/ 100	1 / 2 / 3 / 4 / 5
____/____ D/E	minutes	0/ 25/ 50/ 75/ 100	0/ 25/ 50/ 75/ 100	0/ 25/ 50/ 75/ 100	1 / 2 / 3 / 4 / 5
____/____ D/E	minutes	0/ 25/ 50/ 75/ 100	0/ 25/ 50/ 75/ 100	0/ 25/ 50/ 75/ 100	1 / 2 / 3 / 4 / 5
____/____ D/E	minutes	0/ 25/ 50/ 75/ 100	0/ 25/ 50/ 75/ 100	0/ 25/ 50/ 75/ 100	1 / 2 / 3 / 4 / 5

Have you practiced any mindfulness on your own this week? **Yes / No** (if yes please detail below)

Any comments:

____
____
____
____

Study # ____

Mindful Kangaroo Care: Mindfulness Intervention for mothers during skin-to-skin care: a randomized control pilot study.
Additional file #7: Kangaroo Care Log Control Group

Week 2:

Please write date and circle D for day time (7am-4pm), E for evening (after 4pm)
Please write approximately how long in minutes lasted the kangaroo care

Circle approximately how much time you spent 1- using your smartphone, 2- sleeping and 3-engaged in a conversation with people around you during kangaroo care (0% - 25% - 50% - 75% or 100%)

Last question (How satisfied ...): Circle 1= Very dissatisfied, 2= Slightly dissatisfied, 3= Neutral, 4= Slightly Satisfied, 5= Very satisfied

Date & Time (Month/Day) Day/Evening	Kangaroo Care Duration in minutes (approx)	Smartphone for how much time? From 0-100%	Sleeping for how much time? From 0-100%	Conversation for how much time? From 0-100%	How satisfied were you after this Kangaroo care?
____/____ D/E	minutes	0/ 25/ 50/ 75/ 100	0/ 25/ 50/ 75/ 100	0/ 25/ 50/ 75/ 100	1 / 2 / 3 / 4 / 5
____/____ D/E	minutes	0/ 25/ 50/ 75/ 100	0/ 25/ 50/ 75/ 100	0/ 25/ 50/ 75/ 100	1 / 2 / 3 / 4 / 5
____/____ D/E	minutes	0/ 25/ 50/ 75/ 100	0/ 25/ 50/ 75/ 100	0/ 25/ 50/ 75/ 100	1 / 2 / 3 / 4 / 5
____/____ D/E	minutes	0/ 25/ 50/ 75/ 100	0/ 25/ 50/ 75/ 100	0/ 25/ 50/ 75/ 100	1 / 2 / 3 / 4 / 5
____/____ D/E	minutes	0/ 25/ 50/ 75/ 100	0/ 25/ 50/ 75/ 100	0/ 25/ 50/ 75/ 100	1 / 2 / 3 / 4 / 5
____/____ D/E	minutes	0/ 25/ 50/ 75/ 100	0/ 25/ 50/ 75/ 100	0/ 25/ 50/ 75/ 100	1 / 2 / 3 / 4 / 5
____/____ D/E	minutes	0/ 25/ 50/ 75/ 100	0/ 25/ 50/ 75/ 100	0/ 25/ 50/ 75/ 100	1 / 2 / 3 / 4 / 5
____/____ D/E	minutes	0/ 25/ 50/ 75/ 100	0/ 25/ 50/ 75/ 100	0/ 25/ 50/ 75/ 100	1 / 2 / 3 / 4 / 5

Have you practiced any mindfulness on your own this week? **Yes / No** (if yes please detail below)

Any comments:

____
____
____
____

Study # ____

Mindful Kangaroo Care: Mindfulness Intervention for mothers during skin-to-skin care: a randomized control pilot study.
Additional file #7: Kangaroo Care Log Control Group

Week 3:

Please write date and circle D for day time (7am-4pm), E for evening (after 4pm)
Please write approximately how long in minutes lasted the kangaroo care

Circle approximately how much time you spent 1- using your smartphone, 2- sleeping and 3-engaged in a conversation with people around you during kangaroo care (0% - 25% - 50% - 75% or 100%)

Last question (How satisfied ...): Circle 1= Very dissatisfied, 2= Slightly dissatisfied, 3= Neutral, 4= Slightly Satisfied, 5= Very satisfied

Date & Time (Month/Day) Day/Evening	Kangaroo Care Duration in minutes (approx)	Smartphone for how much time? From 0-100%	Sleeping for how much time? From 0-100%	Conversation for how much time? From 0-100%	How satisfied were you after this Kangaroo care?
____/____ D/E	minutes	0/ 25/ 50/ 75/ 100	0/ 25/ 50/ 75/ 100	0/ 25/ 50/ 75/ 100	1 / 2 / 3 / 4 / 5
____/____ D/E	minutes	0/ 25/ 50/ 75/ 100	0/ 25/ 50/ 75/ 100	0/ 25/ 50/ 75/ 100	1 / 2 / 3 / 4 / 5
____/____ D/E	minutes	0/ 25/ 50/ 75/ 100	0/ 25/ 50/ 75/ 100	0/ 25/ 50/ 75/ 100	1 / 2 / 3 / 4 / 5
____/____ D/E	minutes	0/ 25/ 50/ 75/ 100	0/ 25/ 50/ 75/ 100	0/ 25/ 50/ 75/ 100	1 / 2 / 3 / 4 / 5
____/____ D/E	minutes	0/ 25/ 50/ 75/ 100	0/ 25/ 50/ 75/ 100	0/ 25/ 50/ 75/ 100	1 / 2 / 3 / 4 / 5
____/____ D/E	minutes	0/ 25/ 50/ 75/ 100	0/ 25/ 50/ 75/ 100	0/ 25/ 50/ 75/ 100	1 / 2 / 3 / 4 / 5
____/____ D/E	minutes	0/ 25/ 50/ 75/ 100	0/ 25/ 50/ 75/ 100	0/ 25/ 50/ 75/ 100	1 / 2 / 3 / 4 / 5
____/____ D/E	minutes	0/ 25/ 50/ 75/ 100	0/ 25/ 50/ 75/ 100	0/ 25/ 50/ 75/ 100	1 / 2 / 3 / 4 / 5

Have you practiced any mindfulness on your own this week? **Yes / No** (if yes please detail below)

Any comments:
____
____
____
____

Study # ____

Mindful Kangaroo Care: Mindfulness Intervention for mothers during skin-to-skin care: a randomized control pilot study.
Additional file #7: Kangaroo Care Log Control Group

Week 4:

Please write date and circle D for day time (7am-4pm), E for evening (after 4pm)
Please write approximately how long in minutes lasted the kangaroo care

Circle approximately how much time you spent 1- using your smartphone, 2- sleeping and 3-engaged in a conversation with people around you during kangaroo care (0% - 25% - 50% - 75% or 100%)

Last question (How satisfied ...): Circle 1= Very dissatisfied, 2= Slightly dissatisfied, 3= Neutral, 4= Slightly Satisfied, 5= Very satisfied

Date & Time (Month/Day) Day/Evening	Kangaroo Care Duration in minutes (approx)	Smartphone for how much time? From 0-100%	Sleeping for how much time? From 0-100%	Conversation for how much time? From 0-100%	How satisfied were you after this Kangaroo care?
____/____ D/E	minutes	0/ 25/ 50/ 75/ 100	0/ 25/ 50/ 75/ 100	0/ 25/ 50/ 75/ 100	1 / 2 / 3 / 4 / 5
____/____ D/E	minutes	0/ 25/ 50/ 75/ 100	0/ 25/ 50/ 75/ 100	0/ 25/ 50/ 75/ 100	1 / 2 / 3 / 4 / 5
____/____ D/E	minutes	0/ 25/ 50/ 75/ 100	0/ 25/ 50/ 75/ 100	0/ 25/ 50/ 75/ 100	1 / 2 / 3 / 4 / 5
____/____ D/E	minutes	0/ 25/ 50/ 75/ 100	0/ 25/ 50/ 75/ 100	0/ 25/ 50/ 75/ 100	1 / 2 / 3 / 4 / 5
____/____ D/E	minutes	0/ 25/ 50/ 75/ 100	0/ 25/ 50/ 75/ 100	0/ 25/ 50/ 75/ 100	1 / 2 / 3 / 4 / 5
____/____ D/E	minutes	0/ 25/ 50/ 75/ 100	0/ 25/ 50/ 75/ 100	0/ 25/ 50/ 75/ 100	1 / 2 / 3 / 4 / 5
____/____ D/E	minutes	0/ 25/ 50/ 75/ 100	0/ 25/ 50/ 75/ 100	0/ 25/ 50/ 75/ 100	1 / 2 / 3 / 4 / 5
____/____ D/E	minutes	0/ 25/ 50/ 75/ 100	0/ 25/ 50/ 75/ 100	0/ 25/ 50/ 75/ 100	1 / 2 / 3 / 4 / 5

Have you practiced any mindfulness on your own this week? **Yes / No** (if yes please detail below)

Any comments:
____
____
____
____
