# Supplementary material for: Mindful Kangaroo Care: mindfulness intervention for mothers during skin-to-skin care: a randomized control pilot study
Source: BMC Pregnancy Childbirth. 2022 Jan 15;22:35. doi: 10.1186/s12884-021-04336-w (PMC8761274; doi:10.1186/s12884-021-04336-w)
Supplement: Supplementary file 8 — Additional file 8. Acceptability form. 3-point Likert scale created by the research team to measure the acceptability of the different instruments and mindfulness intervention completed by the mothers at the end of the study. [file 12884_2021_4336_MOESM8_ESM.pdf]

Study # ____

Mindful Kangaroo Care: Mindfulness Intervention for mothers during skin-to-skin care: a randomized control pilot study.

Additional file #8: Acceptability form, both groups

Thanks for participating in our study.

We would like to ask **how acceptable** to you were the different interventions that we used during this research project.

Please place an X under the appropriate column			
	Unacceptable*	Neutral	Acceptable
General Demographic Form (1st week)			
Mindfulness instructions (live)<br>(if applicable)			
Mindfulness audio recordings<br>(if applicable)			
Weekly visit with one of the team member			
Kangaroo care log (weekly)			
Mindfulness scale (13 questions)<br>(1st and last week)			
Parent Stress scale (45 questions)<br>(1st and last week)			
Depression and Anxiety scale (4 quest)<br>(1st and last week)			

* For each unacceptable, please provide some comments to help us to improve this research study:

____
____
____
____
____
____
____
____

Study # ____

Mindful Kangaroo Care: Mindfulness Intervention for mothers during skin-to-skin care: a randomized control pilot study.

Additional file #8: Acceptability form, intervention group only

Any comments about your mindfulness experience. How was it like for you? What did you like? How did it change your experience of the NICU? What was the impact of practicing mindfulness in your experience?
