# Supplementary material for: Mindful Kangaroo Care: mindfulness intervention for mothers during skin-to-skin care: a randomized control pilot study
Source: BMC Pregnancy Childbirth. 2022 Jan 15;22:35. doi: 10.1186/s12884-021-04336-w (PMC8761274; doi:10.1186/s12884-021-04336-w)
Supplement: Supplementary file 9 — Additional file 9. Feasibility form. Tool created by the research team, filled out during the study for every mother recruited after each step of the research project with the following components: recruitment capability, data collection procedures and outcome measures, resources and ability to implement the study. [file 12884_2021_4336_MOESM9_ESM.pdf]

Study # _____

Mindful Kangaroo Care: Mindfulness Intervention for mothers during skin-to-skin care: a randomized control pilot study.

Additional file #9: Feasibility form

Recruitment capability

Date recruited (D/M/Y) : ____ / ____ / 2019

Who recruited: ______________________________

Completed the study (4 weeks): Yes / No ; if no, how many weeks ________

Comments: ____

____

____

Data collection (0= not completed, 1= partially completed, 2= fully completed)

General Demographic Form: ____

Week 1 Kangaroo care log: ____

Week 2 Kangaroo care log: ____

Week 3 Kangaroo care log: ____

Week 4 Kangaroo care log: ____

Week 1 Mindfulness scale: ____

Week 4 Mindfulness scale: ____

Week 1 Stress scale: ____

Week 4 Stress scale: ____

Week 1 Depression and Anxiety: _____

Week 4 Depression and Anxiety: _____

Acceptability form: _____

Comments: ____

____

____

Study # _____

Resources

Week 1 duration of encounter (in minutes) ________ min

Week 1 easiness to meet the patient in kangaroo care (intervention) or meet patient (control)

(1= Complicated, 2= Neutral, 3= Easy)

Availability of investigator to coach: (0=None, 1 = Only One, 2= More than one)

Comments: ____

Week 2 duration of encounter (in minutes) ________ min

Week 2 easiness to meet the patient in kangaroo care (intervention) or meet patient (control)

(1= Complicated, 2= Neutral, 3= Easy)

Availability of investigator to coach: (0=None, 1 = Only One, 2= More than one)

Comments: ____

Week 3 duration of encounter (in minutes) ________ min

Week 3 easiness to meet the patient (1= Complicated, 2= Neutral, 3= Easy)

Availability of investigator to coach: (0=None, 1 = Only One, 2= More than one)

Comments: ____

Week 4 duration of encounter (in minutes) ________ min

Week 4 easiness to meet the patient (1= Complicated, 2= Neutral, 3= Easy)

Availability of investigator to coach: (0=None, 1 = Only One, 2= More than one)

Comments: ____

Issues to download or stream 1st week MP3 recording (intervention only): _________

Issues to download or stream 2nd week MP3 recording (intervention only): _________

Comments: ____

____

____
